# PREDICTORS OF DECISION MAKING FOR AGING-IN-PLACE SUPPORT IN ALZHEIMER’S DISEASE

**DOI:** 10.1093/geroni/igad104.2640

**Published:** 2023-12-21

**Authors:** Amber Miller-Winder, Raven Relerford, Allie Schierer, Charles Olvera, Alaine Murawski, Vanessa Ramirez-Zohfeld, Lee A Lindquist

**Affiliations:** Northwestern University, Chicago, Illinois, United States; Northwestern University, Chicago, Illinois, United States; Northwestern University, Chicago, Illinois, United States; Northwestern University, Chicago, Illinois, United States; Northwestern University, Chicago, Illinois, United States; Northwestern University, Chicago, Illinois, United States; Northwestern University, Chicago, Illinois, United States

## Abstract

Older adults with Alzheimer’s Disease (AD) need additional support in their lifetime, yet little is known about how the decision to accept help is made. Our goal is to better understand how older adults’ aging-in-place (AIP) decision making is impacted by worsening cognition, functional loss, social influences, and environmental factors. This longitudinal study consists of older adults from an NIA-funded cohort (LitCog) with extensive cognitive testing. The intervention, PlanYourLifespan.org (PYL), facilitates making decisions about AIP needs, including AD. After viewing PYL, subjects are surveyed every 6 months with cognitive, social, functional, health literacy, environmental and decision-making variables. Of 293 subjects, (mean age 73.5, 72.7% female, 40.4% under-represented minority) almost half (47.4%) experienced cognitive decline with 10.3% identifying worsening memory loss from the prior timepoint. Subjects were asked: If you developed AD, have you decided your living/support preferences? At 1-mo, subjects were significantly more likely to have made decisions if they had: limited health literacy (OR 4.36 [p< 0.01, 1.69-11.24]), larger social networks (OR 1.08 [p< 0.05, 1.01-1.15]), completion of a living will (OR 2.43 [p< 0.05,1.11-5.33]. At 6-mo, sufficient social support (OR 3.39 [p< 0.05, 1.19-9.70]. At 12-mo, higher social isolation (OR 1.05 [p< 0.05, 1.01-1.08]) and self-efficacy (OR 1.07 [p< 0.01, 1.04-1.11]). In the event of worsening cognition, the likelihood of making care decision is associated with external (social support) and internal (self-efficacy, health literacy) variables, which change in significance over time. Catastrophic factors (COVID-19) impacted comfortability of utilizing long-term care facilities.

